# Functionalization of zirconia ceramic with fibronectin proteins enhanced bioactivity and osteogenic response of osteoblast-like cells

**DOI:** 10.3389/fbioe.2023.1159639

**Published:** 2023-04-25

**Authors:** Lwin Moe Aung, Jerry Chin-Yi Lin, Eisner Salamanca, Yi-Fan Wu, Yu-Hwan Pan, Nai-Chia Teng, Haw-Ming Huang, Ying-Sui Sun, Wei-Jen Chang

**Affiliations:** ^1^ School of Dentistry, College of Oral Medicine, Taipei Medical University, Taipei, Taiwan; ^2^ Department of Oral Medicine, Infection and Immunity, Harvard School of Dental Medicine, Boston, MA, United States; ^3^ Department of Dentistry, Chang Gung Memorial Hospital, Taipei, Taiwan; ^4^ Graduate Institute of Dental and Craniofacial Science, Chang Gung University, Taoyuan, Taiwan; ^5^ School of Dentistry, College of Medicine, China Medical University, Taichung, Taiwan; ^6^ Department of Dentistry, Taipei Medical University Hospital, Taipei, Taiwan; ^7^ School of Dental Technology, College of Oral Medicine, Taipei Medical University, Taipei, Taiwan; ^8^ Dental Department, Shuang-Ho Hospital, Taipei Medical University, New Taipei City, Taiwan

**Keywords:** zirconia, glow discharge plasma, surface treatment, allylamine, fibronectin

## Abstract

**Introduction:** To overcome the genuine bioinert properties of zirconia ceramic, functionalization of the surface with the bioactive protein fibronectin was conducted.

**Methods:** Glow discharge plasma (GDP)-Argon was first used to clean the zirconia surface. Then allylamine was treated at three different powers of 50 W, 75 W, and 85 W and immersed into 2 different fibronectin concentrations (5 µg/ml and 10 µg/ml).

**Results and Discussion:** After surface treatment, irregularly folded protein-like substances were attached on the fibronectin coated disks, and a granular pattern was observed for allylamine grafted samples. Infrared spectroscopy detected C-O, N-O, N-H, C-H, and O-H functional groups for fibronectin treated samples. Surface roughness rose and hydrophilicity improved after the surface modification, with MTT assay showing the highest level of cell viability for the A50F10 group. Cell differentiation markers also showed that fibronectin grafted disks with A50F10 and A85F10 were the most active, which in turn encouraged late-stage mineralization activity on 21d. Up-regulation of osteogenic related mRNA expression from 1d to 10d can be observed in RT-qPCR data for ALP, OC, DLX5, SP7, OPG and RANK biomarkers. These physical and biological properties clearly indicate that an allylamine and fibronectin composite grafted surface significantly stimulated the bioactivity of osteoblast-like cells, and can be utilized for future dental implant applications.

## 1 Introduction

Dental implants are a valuable treatment option for oral rehabilitation that have been used to replacing missing teeth since the 1960 s. Recently, traditional titanium and titanium alloy implants have been criticized for their lack of esthetic appeal and the release into the oral environment of titanium and other elemental ions that can lead to biological complications (Mombelli et al., 2018). This has prompted the adoption of zirconium oxide or zirconia ceramic material as a promising biomaterial for crowns, onlays, inlays, implant abutments, and fixtures over the last decade. Zirconia (Zr) mimics the natural color of teeth and has high chemical stability (Yin et al., 2017; Zhang and Lawn, 2018). Furthermore, it displays unique mechanical properties such as high fracture resistance, high bending strength, high corrosion resistance, and radiopacity. Lower bacterial adhesion reduces the likelihood of inflammation in peri-implant tissues, and also encourages the usage of zirconia ceramic (Bona et al., 2015; Borgonovo et al., 2021).

Nevertheless, the bioinert nature of zirconia and its pure surface hinders the interaction with surrounding osteoblasts cells (Hanawa, 2020). Researchers have thus sought to enhance surface properties and interaction with surrounding bone cells by developing various surface treatment techniques, such as sand-blasting, acid-etching, anodization, additive sintering, and laser structuring (Pieralli et al., 2018). One recent development is the creation of a biologic environment via the grafting of the biologically active protein fibronectin onto implant surfaces through glow discharge plasma (GDP) treatment (Pan et al., 2020).

GDP treatment is widely used to sterilize and modify the surface of biomaterials. This method utilizes low thermal plasma and low atmospheric pressure to form functional proteins (Canullo et al., 2016). This process was developed to enable the surface treatment of biodegradable polymers without altering the original material’s bulk properties (Yang et al., 2015). Most importantly, GDP treatment in combination with allylamine and fibronectin is used to improve the wettability and hydrophilicity of zirconia surfaces. In addition, this technique has been regarded as an efficient tool to optimize the biocompatibility of biomaterials in dentistry (Chang et al., 2015). Allylamine (A) is a plasma precursor used to generate amine functionalities on biomaterials to facilitate the bonding of biologically active proteins on surfaces (Rohr et al., 2020). The polar groups associated with a nitrogen-rich surface chemistry have been shown to promote cellular attachment (Sharifahmadian et al., 2021) while the positive charge of amine groups attracts negatively charged biomolecules, which thereby provides an ideal connection between the material and cells (Buddhadasa et al., 2018).

Fibronectin (F) is an extracellular matrix (ECM) protein with a high molecular weight (440 and 500 kD) that binds to specific cell membrane receptors referred to as integrins. The presence of fibronectin is highly favorable to material-cell interactions in terms of cell adhesion, migration, and proliferation (Pankov and Yamada, 2002). Fibronectin exists as a protein dimer comprising two monomers linked by a pair of disulfide bonds. One monomer binds to cells while the other binds to ECM proteins such as collagen, fibrin, and heparan sulfate proteoglycans. Fibronectin plays a pivotal role in cell-to-cell and cell-to-substrate adhesion as well as an essential role interacting with surrounding bone cells due to its ability to allow osteoblast attachment to ECM components (Dalton and Lemmon, 2021). The adhesion of cells to substrates strongly depends on fibronectin, and fibronectin enhances fibroblast proliferation and osteogenic activity, leading to improved healing after implant surgery and during the maintenance phase (Zhang et al., 2017). Fibronectin is also proven to enhance cell adhesion, differentiation, and bone regeneration on fibronectin-linked titanium surfaces (Shibata et al., 2002; Park et al., 2021). However, there has been relatively little research on the use of bioactive fibronectin on zirconia ceramic biomaterials, and, to our knowledge, the efficacy of fibronectin on zirconia ceramic surfaces modified by using GDP has yet to be verified. This study was therefore conducted to investigate the novel properties and reaction of allylamine-fibronectin composite grafting on surface of zirconia ceramic and with surrounding bone cells.

## 2 Materials and methods

### 2.1 Sample preparation

#### 2.1.1 Zirconia disks preparation

Zr disks (Coho Technology Co. Ltd., Taipei, Taiwan; diameter: 10 mm and thickness: 1 mm) were immersed in a detergent solution and subjected to ultrasonic cleansing, followed by rinsing with distilled water. The disks were then submerged in acetone, followed by two washes with distilled water. Finally, the Zr disks were sterilized in an autoclave at 121°C for 30 min and dried at 40°C in a conventional oven.

#### 2.1.2 Protein grafting

**FIGURE 1:**
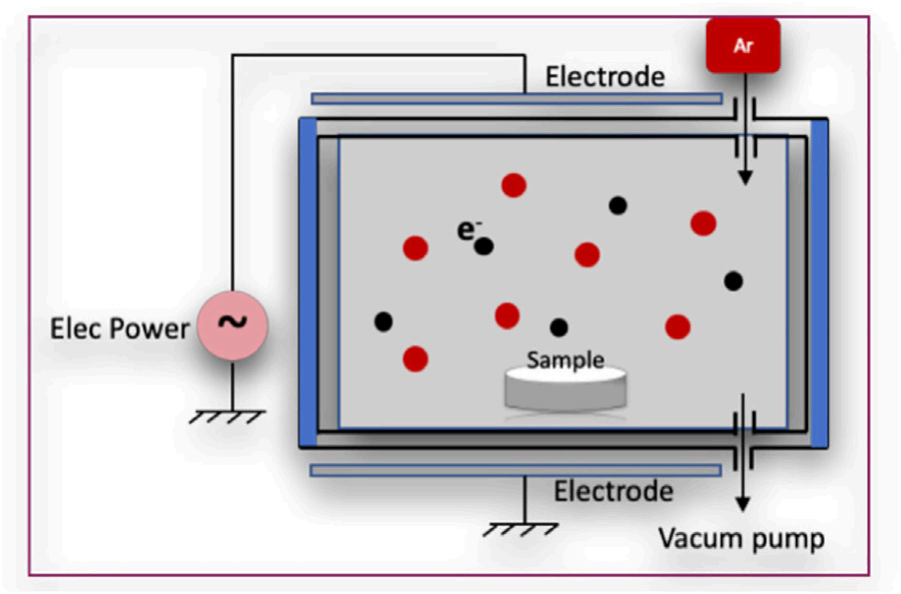
Glow discharge plasma reactor.

After argon plasma treatment, specimens were subjected to the Allylamine organic compound in a GDP machine (Figure 1), at powers of 50 W, 75 W, or 85 W with 13.56 MHz and 100 millitorrs for 30 min. The samples with allylamine were labeled according to power as follows: A50, A75, and A85.

**FIGURE 2:**
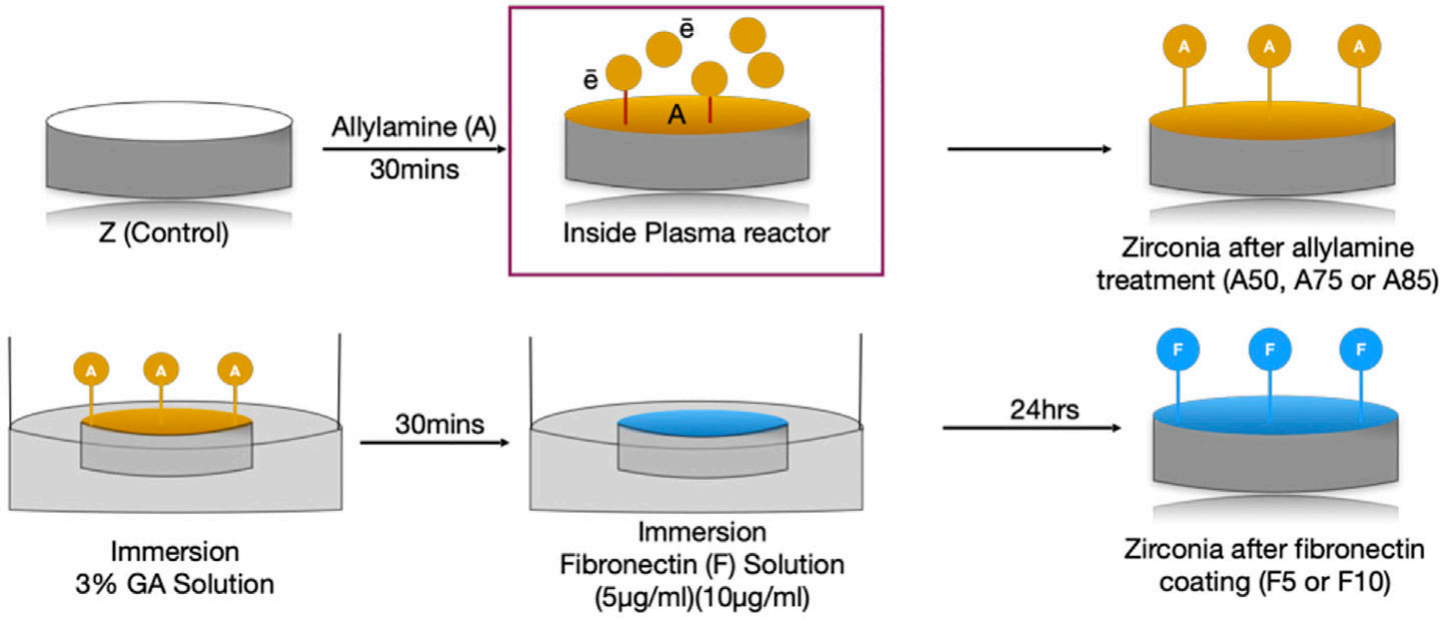
Illustration of flowchart of sample preparation and protein grafting.

Specimens were then immediately immersed in a 3% glutaraldehyde (GA) solution (Merck, NJ, United States) for 30 min to facilitate a chain reaction between allylamine (A) and fibronectin (F). After two rounds of rinsing with 0.1 M phosphate-buffered saline (PBS, Wako Pure Chemical Industries, Osaka, Japan), the zirconia disks were dipped in fibronectin solutions at concentrations of 5 μg/mL or 10 μg/mL (Sigma-Aldrich Co., St. Louis, MO, United States) for 24 h to graft the surface with fibronectin. Finally, specimens were submerged in Tris-phosphate buffer solution (2-amino-2-hydroxymethyl-1, 3-propandiol 999, Wako Pure Chemical Industries, Osaka, Japan) (pH 7.4) for 30 min. The Zr disks were labeled according to processing condition as follows: A50F5, A50F10, A75F5, A75F10, A85F5, and A85F10 (Figure 2).

### 2.2 Surface morphology observation

In this study, all zirconia disks were coated with nanogold particles first and placed in a specimen stub for SEM use (SU-3500; Hitachi Ltd., Kyoto, Japan). Analysis was then performed using an accelerating voltage of 20 kV in vacuum mode and an electron image was obtained. Four random disk areas were captured at ×200 and ×2000 magnification to observe surface morphology. Analysis with performed with the default Hitachi PC SEM software provided by the factory attached to the original machine.

### 2.3 Surface element identification by X-ray photoelectron spectroscopy analysis (XPS)

X-ray photoelectron spectroscopy (XPS) (PHI Quantera II, Japan) was used to acquire Al Kα or Mg Kα excitation (15 kV, 20 mA) at a take-off angle of 15° relative to the normal sample. Analyzing conditions were 200 µm X-ray spot size and a 2 h charge survey electron flood gun was utilized. The energies and intensities of the photoelectron peaks enable the identification and quantification of surface elements including C1s and N1s. SmartSoft-XPS software was utilized for data analysis.

### 2.4 Functional group analysis (ATR-FTIR)

ATR-FTIR for all sample groups was performed using a Nicolet iS5 (Thermo Fisher Scientific, Madison, WI, United States) equipped with an iD7 crystal ZnSe in reflection mode. The absorbance spectra of the control and fibronectin grafted specimens were measured using 16 scans coded with a 0.482 cm^−1^ resolution. This procedure provides information related to the presence or absence of specific functional groups and the chemical structure of polymer materials. FITR spectra in the region of 4,000–650 cm^−1^ was obtained, as well as a background spectrum used for normalization. The absorbance of spectra is measured, and atomic peaks can be obtained. As zirconia disks are opaque, measurements were made using semi-attenuated total reflection (ATR).

### 2.5 Immunofluorescence assay by fluorescein-5-isothiocyanate (FITC) labeling

Fluorescein isothiocyanate (FITC) labeling was used to observe the amount of fibronectin absorbed on fibronectin grafted samples. To evaluate the concentration difference between 5 μg/mL and 10 μg/mL and to determine the concentration of fibronectin attachment on the zirconia surface, fluorescein isothiocyanate (FITC) labeling was performed. FITC reagent (Sigma-Aldrich Co., St. Louis, MO, United States) was prepared according to manufacturer’s instructions. Fibronectin-coated disks were fixed with formaldehyde and washed with PBS and exposed to a blocking buffer for 10 min. Then disks were immersed in the FITC solution for 24 h and fibronectin dots on the zirconia surfaces were observed using a confocal laser microscope (Leica Stellaris 8, Germany).

### 2.6 Surface wettability analysis

Wettability was measured via static water contact angles on the Zr disks for both groups using a 35-mm camera. A 4 μL water droplet (Millipore-Q, Millipore, Bedford, MA, United States; filtered, 20°C) was set on top of each surface and the contact angles of each experimental sample were measured.

### 2.7 Surface roughness analysis by optical profiler using white light interferometer (WLI)

All surface roughness measurements were performed with an Optical Profilometer (OP) from Bruker Contour GT-K Elite (Billerica, Massachusetts, United States) using the White Light Interferometer (WLI). The reference beam in the machine is reflected by the reference mirror, while the measurement beam is reflected or scattered from the test surface. Surface roughness (Ra) was measured for the Zr disk surfaces. The program was set according to the manufacturer’s protocol and measurements of each specimen conducted over a 128 μm × 96 µm scan area at ×50 magnification along the *X* and *Y* direction. Using a maximum scan speed of 47 μm/sec (with standard camera) and 0.05%–100% sample reflectivity. Vision64 data analyzer software is used in combination with the hardware.

### 2.8 Biological responses

#### 2.8.1 Cell culturing

MG-63 cells were obtained from the Cell Cultures of the Bioresource Collection and Research Center Collection (Hsinchu, Taiwan). MG-63 cells were maintained in Dulbecco’s modified Eagle’s medium (DMEM; HyClone Laboratories Inc., Logan, UT, United States) withpenicillin–streptomycin (1%), fetal bovine serum (10%), and L-glutamine (4 mmol/L) under humidification with 5% CO_2_ at 37°C.

#### 2.8.2 Fluorescent confocal laser scanning microscopy (CLSM)

Samples including control and fibronectin-coated disks were analyzed for confocal microscopy by using MG-63 osteoblast-like cells. Before starting the microscopy, cells were seeded directly on the sample surface using a cell density of 2.0 × 10^4^ in the 6-well plate and incubated for 12 h and 24 h. After that, culture medium was removed and washed with PBS. Then, cells were fixed on the sample surface with 4% paraformaldehyde at room temperature and left to dry within the laminar flow. Then they were permeabilized with 1% Triton X-100 in PBS for 10 min. After washing three times with 0.1% Triton X-100 in Dulbecco’s PBS, nuclei were stained for 1 h with DAPI (1:1,000 dilution; 5 mg/mL one-stock solution; Sigma-Aldrich), Alexa Fluor 488 phalloidin (1:80 dilution; A12379,c Invitrogen, MA, United States), and stabilized with 10 µL of mounting buffer (Fluoromount-G, Southern Biotech). Finally, confocal fluorescence laser scanning microscopy (CLSM) was conducted (Leica Stellaris 8, Germany) to evaluate adhesion and cell proliferation on the zirconia surface.

#### 2.8.3 Cell viability analysis

On days 1, 3, 5, and 7, cell viability, indirect cell proliferation through metabolic activity, and cytotoxicity for the control, blank control, and test groups were evaluated using a 3-4, 5-dimethylthiazol-2-yl)-2, 5-diphenylt thiazol-2-yl)-2, 5-diphenyltetrazolium bromide (MTT) kit (Roche Applied Science, Mannheim, Germany). Cells cultured on untreated Zr disk surfaces were used as a control because uncoated zirconia ceramic surface can considerably hinder cell proliferation, while cells cultivated on a plate without surface treatment were used as a “blank control” for better analysis. After adding the colorimetric substrate to 6-well plates containing sample disks and incubating them for 4 h at 37°C according to manufacturer instructions. Live cells converted the MTT into a formazan dye, and the addition of dimethyl sulfoxide (DMSO) for 10 min changed the color from yellow to purple, which was quantified using an ELISA reader (SpectraMax iD3 Multi-Mode Microplate Reader from Molecular Devices, United States) at a wavelength of 570 nm.

#### 2.8.4 Alkaline phosphatase enzyme activity assay (ALP)

As ALP production is an indicator of cell differentiation, and an ALPase Activity Assay kit (BioVision, Cat # 412-500, Milpitas, CA, United States) was used to verify osteoblast-like cell differentiation in serum (plasma), tissue, cells, and other samples. Cultured cells were washed with PBS and detached using Triton-100 (300 μL, 0.05%). A measure of 500 µL of assay buffer was used on samples within the 6-well plates and incubated for 2 h. After that, 80 µL of buffer was transferred to a 96-well plate and enzyme activity of cells in contact with control and fibronectin-treated surfaces were analyzed with the same ELISA reader at an optical density (OD) value of 405 nm.

#### 2.8.5 Reverse transcriptase qualitative real-time polymerase chain reaction (RT-qPCR)

Supernatant liquid containing RNA was obtained by harvesting and centrifuging cells using trizol and chloroform. Iso-propranolol was added and centrifuged at 15,000 rpm and 4°C for 30 min. After obtaining messenger RNA (mRNA), concentrations were measured using Nanodrop (Implen Nanophotometer NP-80, Germany). Next, the mRNA was transcribed into cDNA, and qPCR was conducted with Fast SYBRTM Green Master Mix (Thermo Fisher Scientific Baltics UAB, Vilnius, Lithuania) and the LightCycler^®^96 Instrument (Roche Molecular Systems, Inc., Pleasanton, CA, United States). Forward and reverse primer sequences used in this study were designed by Primer-BLAST from the United States (US) National Library of Medicine, as listed in Table 1 (Sollazzo et al., 2010). Quantification was performed utilizing delta–delta calculation.

**TABLE 1:** Forward and reverse primer sequences of gene symbols.

Gene symbol	Forward primer sequence (5′-3′)	Reverse primer sequence (5′-3′)
GAPDH	AAA AAC CTG CCA AAT ATG AT	CAG TGA GGG TCT CTC TCT TC
ALP	CTT GTG CCT GGA CGG ACC CT	TGG TGC ACC CCA AGA CCT GC
OC	GCC​CTC​ACA​CTC​CTC​GCC​CTA​TT	GGG​TCT​CTT​CAC​TAC​CTC​GCT​GCC
DLX5	CAA​CTT​TGC​CCG​AGT​CTT​C	GTT​GAG​AGC​TTT​GCC​ATA​GG
SP7/OSX	TGGCGTCCTCCCTGCTTG	TGC​TTT​GCC​CAG​AGT​TGT​TG
OPG	GAA GGG CGC TAC CTT GAG AT	GCA AAC TGT ATT TCG CTC TGG
RANK	TGT GGC ACT GGA TCA ATG AG	GTC TTG CTG ACC AAT GAG AG

#### 2.8.6 Mineralization analysis by alizarin red S staining

After culturing in medium for 7, 14, and 21 days, cells were washed twice with phosphate-buffered saline and fixed with 70% ethanol for 1 h. After that samples were washed twice with distilled water, stained with 40 mM alizarin red S (Sigma-Aldrich, St. Louis, MO, United States) for 10 min, and finally washed 3 times with distilled water. Finally, 200 µL samples were taken in 96 well plates and checked at OD 540 nm value. The medium was changed every 3 days for all wells to ensure a clean environment and continuous nutrient delivery for the cells.

## 3 Results

### 3.1 Characterization of surface morphology

**FIGURE 3:**
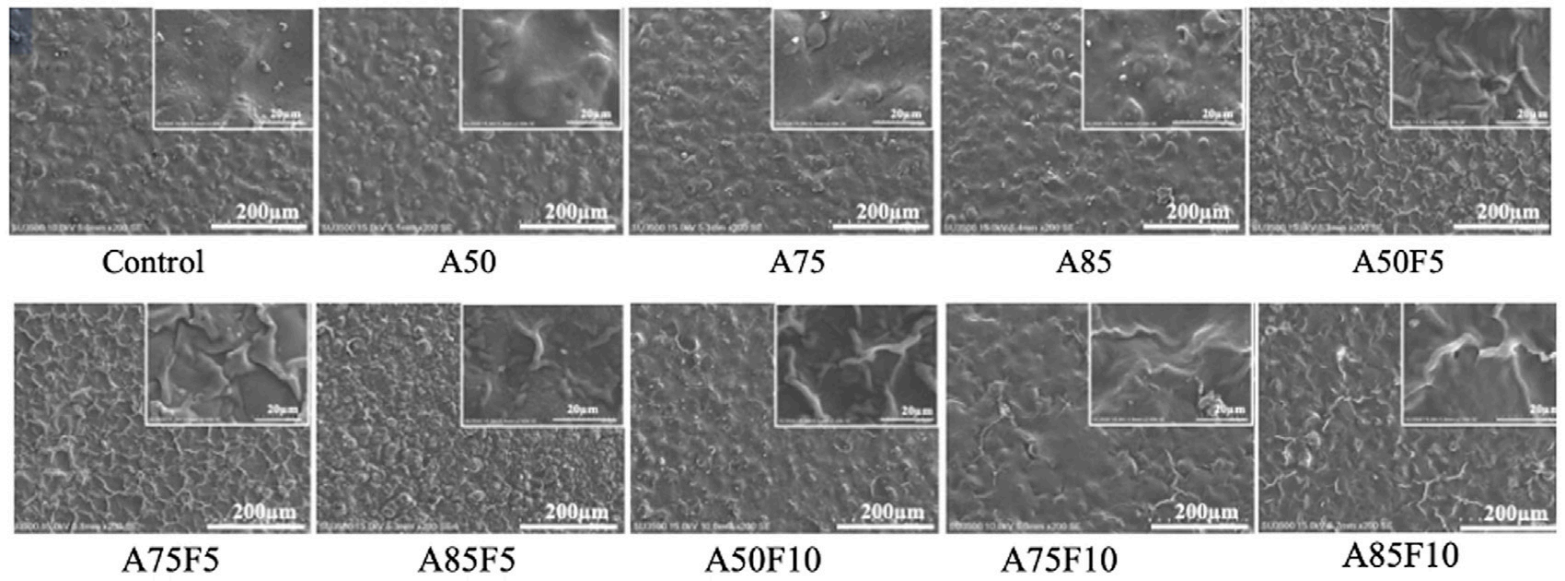
Scanning electron microscopy (SEM) images showing rough granular surfaces on allylamine treated samples and irregular folding proteins on the fibronectin modified surfaces.

Irregular folding of protein-like substances was observed on the surfaces of fibronectin grafted Zr disks (Figure 3). Irregular granular patterns were present on the surface of allylamine-treated samples. SEM images show that as allylamine power increased from A50 to A85, texture patterns and surface deposits became more prominent. Likewise, irregular-folded proteins were more abundant and prominent at a fibronectin concentration of 10 μg/mL than at 5 μg/mL.

### 3.2 Atomic configuration of Zr disks using X-ray photoelectron spectroscopy (XPS)

**FIGURE 4:**
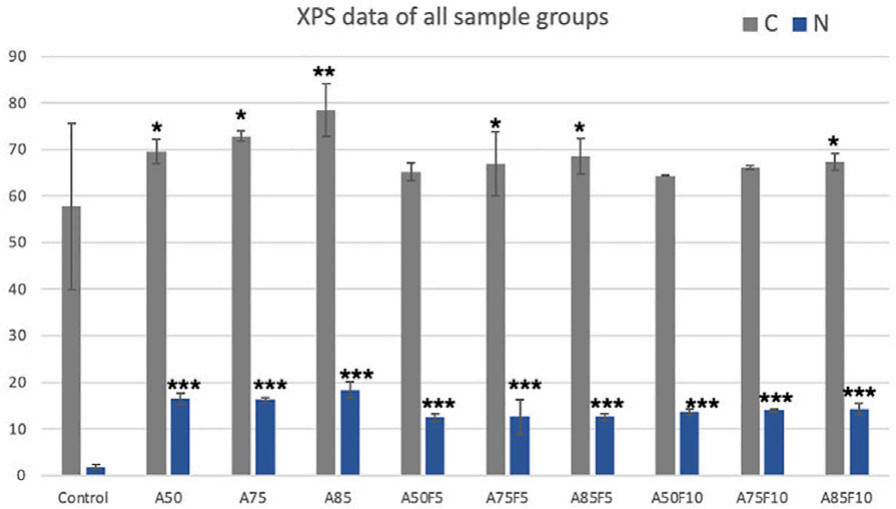
Line graph illustration of XPS data.

XPS data shows that the carbon and nitrogen content of the original zirconia disk was 57.7% (±17.8) and 1.8% (±0.5) respectively (Table 2; Figure 4). All fibronectin treated samples and other groups had higher carbon and nitrogen contents than did the control. The carbon content of the treated zirconia disk was as follows: A50, 69.5 (±2.6); A75, 72.8 (±1.0); A85, 78.4 (±5.7); A50F5, 65.2% (±1.8); A75F5, 66.9% (±6.8); and A85F5, 68.4% (±3.8), A50F10, 64.3 (±0.1); A75F10, 66.1 (±0.3); and A85F10, 67.2 (±1.8).

**TABLE 2:** Elemental composition results by XPS.

Survey	%	%
	C1s	N1s
Control	57.74 (±17.8)	1.81 (±0.51)
A50	69.56 (±2.61)	16.5 (±1.13)
A75	72.83 (±1.09)	17.2 (±0.4)
A85	78.4 (±5.71)	18.39 (±1.68)
A50F5	65.20 (±1.84)	12.53 (±0.80)
A75F5	66.93 (±6.87)	12.58 (±3.8)
A85F5	68.47 (±3.85)	12.6 (±0.72)
A50F10	64.39 (±0.13)	13.69 (±0.53)
A75F10	66.11 (±0.34)	13.98 (±0.25)
A85F10	67.27 (±1.84)	14.33 (±1.06)

Nitrogen content was as follows: A50F5, 12.5% (±0.8); A75F5, 10.3% (±3.8); and A85F5 12.6% (±0.7). Statistically significant differences were found between control, allylamine, and fibronectin-modified disks for both C and N composition. This data trend of the fibronectin groups also matched with data obtained in allylamine-treated groups, i.e., an increase in C and N with an increase in GDP power from A50F5 to A85F10.

### 3.3 Functional groups data by ATR-FTIR

**FIGURE 5:**
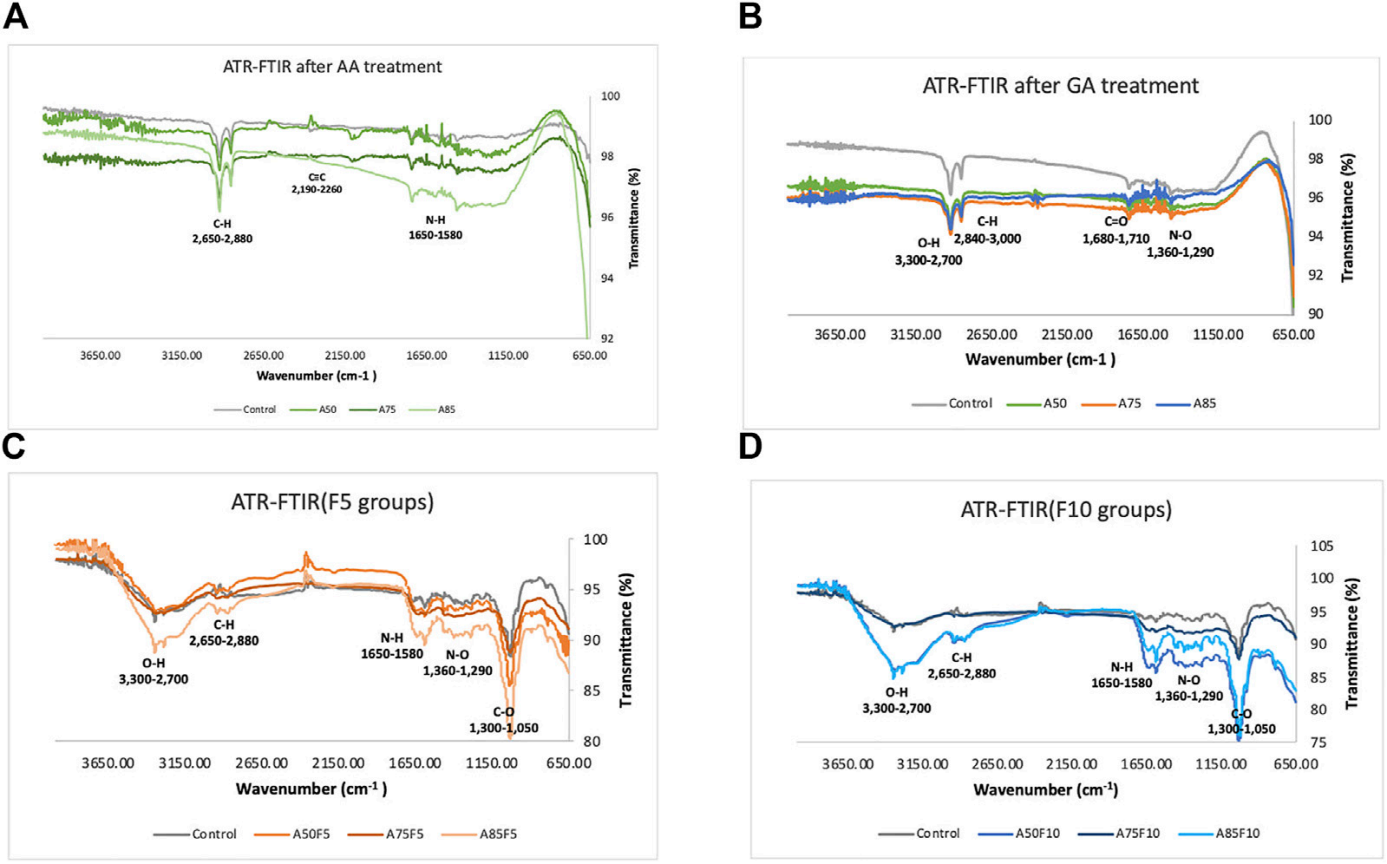
Fourier transformed infrared spectroscopy (FTIR) spectra indicating the atomic peaks and functional groups of **(A)** control and allylamine treated samples, **(B)** control and allylamine groups after GA treatment, **(C)** control and fibronectin 5 µg/mL samples, **(D)** control and fibronectin 10 µg/mL samples.

As concentrations of surface coatings rose, including allylamine and fibronectin, the presence of functional groups became more obvious. Allylamine-treated samples had N-H, C≡C and C-H bonds (Figure 5A) and after crosslinking with glutaraldehyde, N-O, C=O, C-H, and O-H bonds were observed (Figure 5B). Absorption bands rose after fibronectin grafting, with F5 and F10 graphs showing higher C-O, N-O, N-H, C-H, and O-H bonding . C-O, N-H, C-H, and O-H functional bonds were detected for all fibronectin samples. A85F5 and A85F10 groups had the highest number of C-O, C-H and O-H groups, followed by A75F5, A75F10 and A50F5, and A50F10 groups. F5 and F10 groups showed significantly increased C-O, N-H and O-H functional groups after fibronectin grafting (Figures 5C, D). These FTIR functional bonding results complement the XPS elemental data, and taken together these data show the successful functionalization of fibronectin on zirconia ceramic surfaces.

### 3.4 Immunofluorescence assay with FITC labelling

**FIGURE 6:**
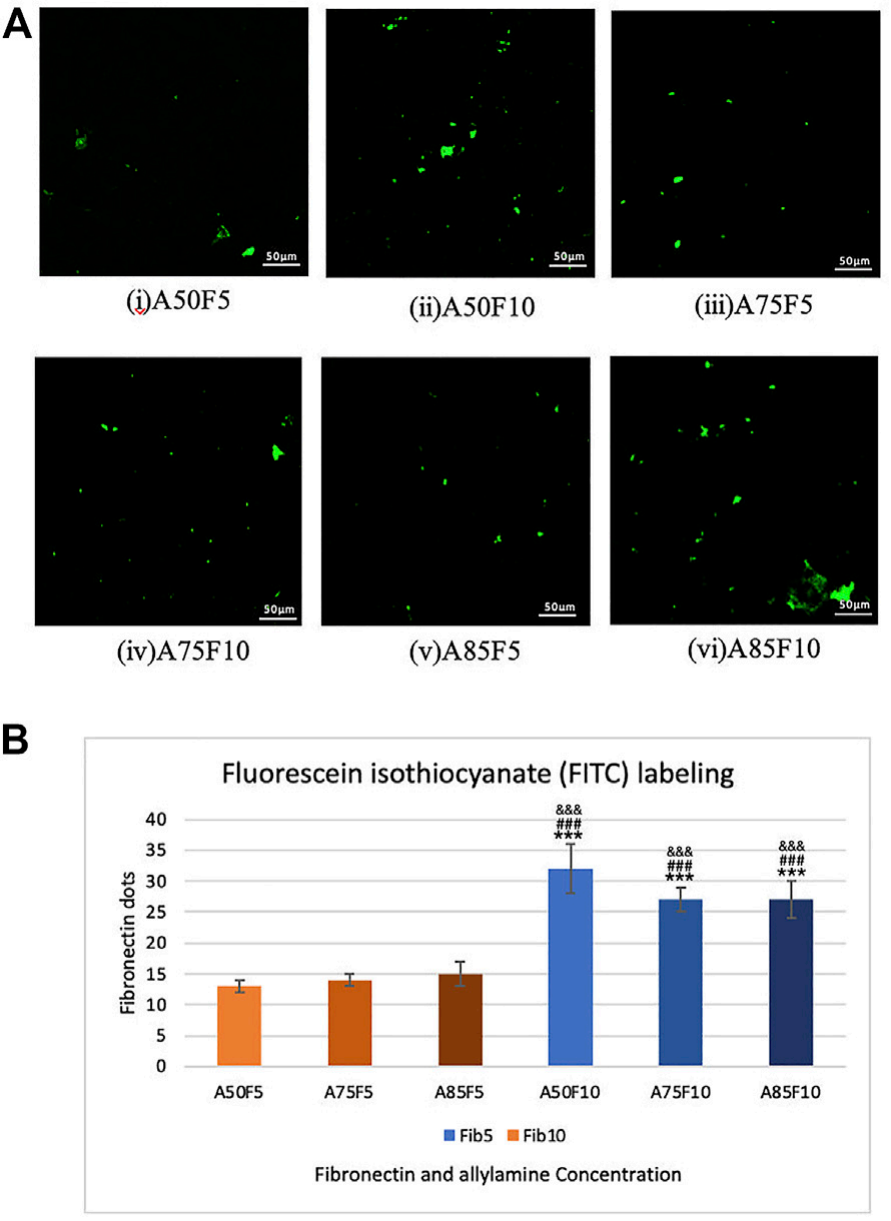
Fibronectin concentration analysis by FIFC labelling images in **(A)**, (i) A50F5, (ii) A50F10. (iii) A75F5. (iv) A75F10, (v) A85F5 and (vi) A85F10 and **(B)** Analysis revealed that the number of fibronectin dots increased with the concentration of fibronectin solution when compared with A50F5 indicated with *, *p* < 0.05, **, *p* < 0.005, and ***, *p* < 0.001, while # symbol for comparing with A75F5 and, & symbol is applied for comparing with A85F5.

The presence of green fibronectin dots or patches confirmed the attachment and adhesion of fibronectin proteins on the surface of zirconia ceramic via confocal microscopy. Differences in the number of dots in concentrations of 5 μg/mL and 10 μg/mL fibronectin can also be seen. The number of positive areas appeared in green and increased with fibronectin concentration as shown in Figure 6A. There is also a statistically significant difference present between inter groups when comparing F5 and F10 in Figure 6B. Because fibronectin is an important determinant of cell adhesion and migration, higher concentrations of fibronectin may also contribute to a quicker cell adhesion and proliferation with zirconia ceramic.

### 3.5 Surface hydrophilicity

**FIGURE 7:**
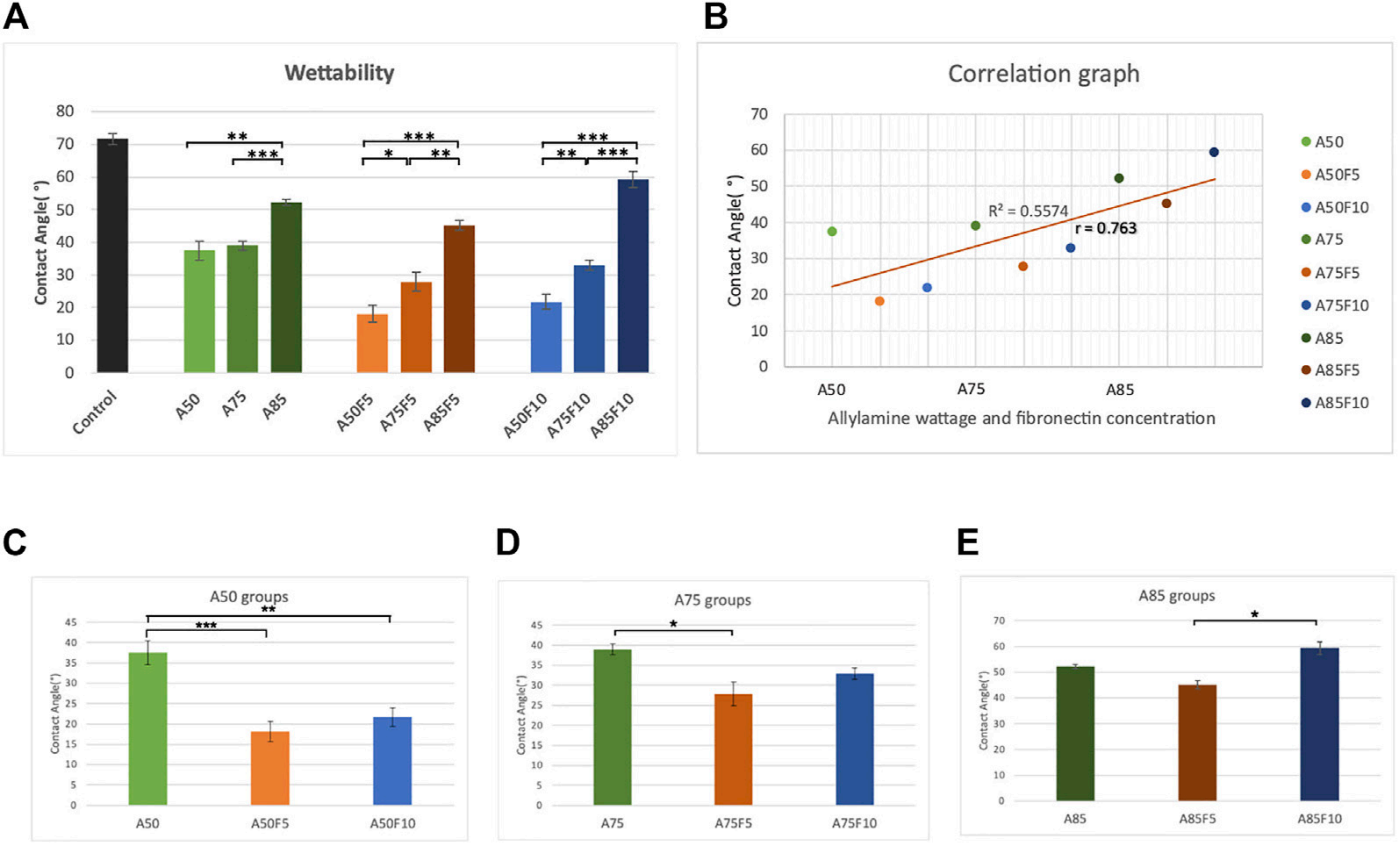
Surface wettability data demonstrating **(A)** better hydrophilicity in fibronectin grafted groups **(B)** strong correlation coefficient between contact angle and concentration of allylamine and fibronectin, surface wettability data of **(C)** control and A50 group **(D)** control and A75 group **(E)** control and A85 group.

The contact angle of water droplets on the surface of untreated original zirconium disk was 71.7 (±1.65°) (Figure 7A). Fibronectin concentration was positively correlated with contact angle; A50F5 (18.1° ± 2.48°), A50F10 (21.7° ± 2.92°), A75F5 (27.8° ± 2.91°), A75F10 (32.9° ± 1.49°), A85F5 (45.1° ± 1.58°), and A85F10 (59.3° ± 2.44°) (Figures 7C–E). Contact angle measurements for the allylamine group are as follows: A50 (37.4° ± 2.92°), A75 (38.9° ± 1.36°), and A85 (52.2° ± 0.81°). After GDP-allylamine and fibronectin treatment, the surface of all sample groups exhibited excellent hydrophilicity and higher surface energy. The contact angle in A85 was about 52.2° and 59.3°in A85F10, but these angles were still smaller than the untreated control disk’s contact angle. A statistically significant difference was found between the fibronectin-grafted groups and other groups, with a varying significance level (*p < 0.05; **p ≤ 0.01; ***p < 0.001). Samples with fibronectin had lower contact angles than those samples with allylamine coating only, indicating that fibronectin improved surface hydrophilicity of zirconia than surfaces more than treatment with allylamine alone.

### 3.6 Surface roughness

**FIGURE 8:**
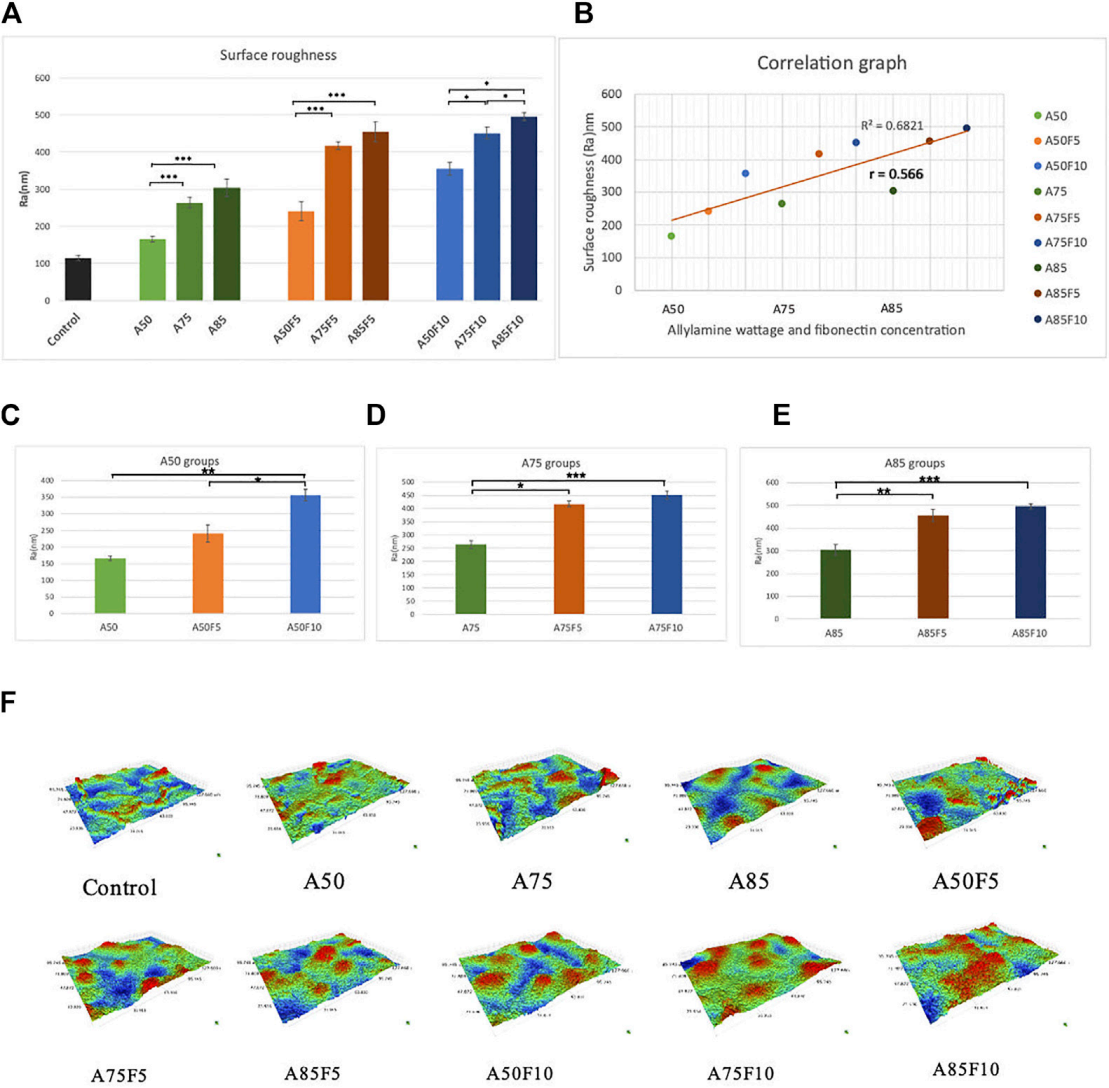
**(A)** Surface roughness analysis showing that increased roughness with allylamine and fibronectin coating **(B)** moderate correlation between increased concentration and surface roughness, surface roughness data of **(C)** control and A50 groups, **(D)** control and A75 groups, **(E)** control and A85 groups, **(F) **surface roughness for each sample in 3-D images.

Fibronectin-grafted zirconia disks demonstrated greater surface roughness than both control and allylamine-treated counterpart disks (Figure 8A). The Ra value of the original zirconia disk was (114.33 ± 6.62 μm) while the roughness value increased in A50 (165.6 ± 7.0 μm), A75 (264 ± 14 μm), and A85 (304 ± 23 μm) after allylamine treatment. After fibronectin grafting, Ra values increased in A50F5 (240 ± 25 μm), A50F10 (355 ± 17 μ), A75F5 (417 ± 11 μm), A75F10 (214 ± 16 μm), A85F5 (455 ± 27 μm), and A85F10 (495 ± 10 μm) (Figures 8C–E). This profilometry analysis surface of roughness falls in line with SEM image analysis (Figure 8F). Student t-test revealed statistically significant differences between fibronectin samples and control (*p < 0.05; **p ≤ 0.01; ***p < 0.001).

### 3.7 Cell viability analysis

One day after cell culturing, the groups showing the highest cell viability were A50F10 and A85F10, followed by A85F5 and A50F5. The only significant difference was seen between experimental groups vs. control and blank control. No significant inter-group difference was present.

On day 3, the A50F10 group had the highest cell viability, followed by A85F10. After 5 days, the cell A50F10 survival rate was still the highest, with other groups following the same pattern of growth with the day 1 and day 3. However, after day 3 and day 5, cell proliferation increased and a significant difference between testing groups was observed.

**FIGURE 9:**
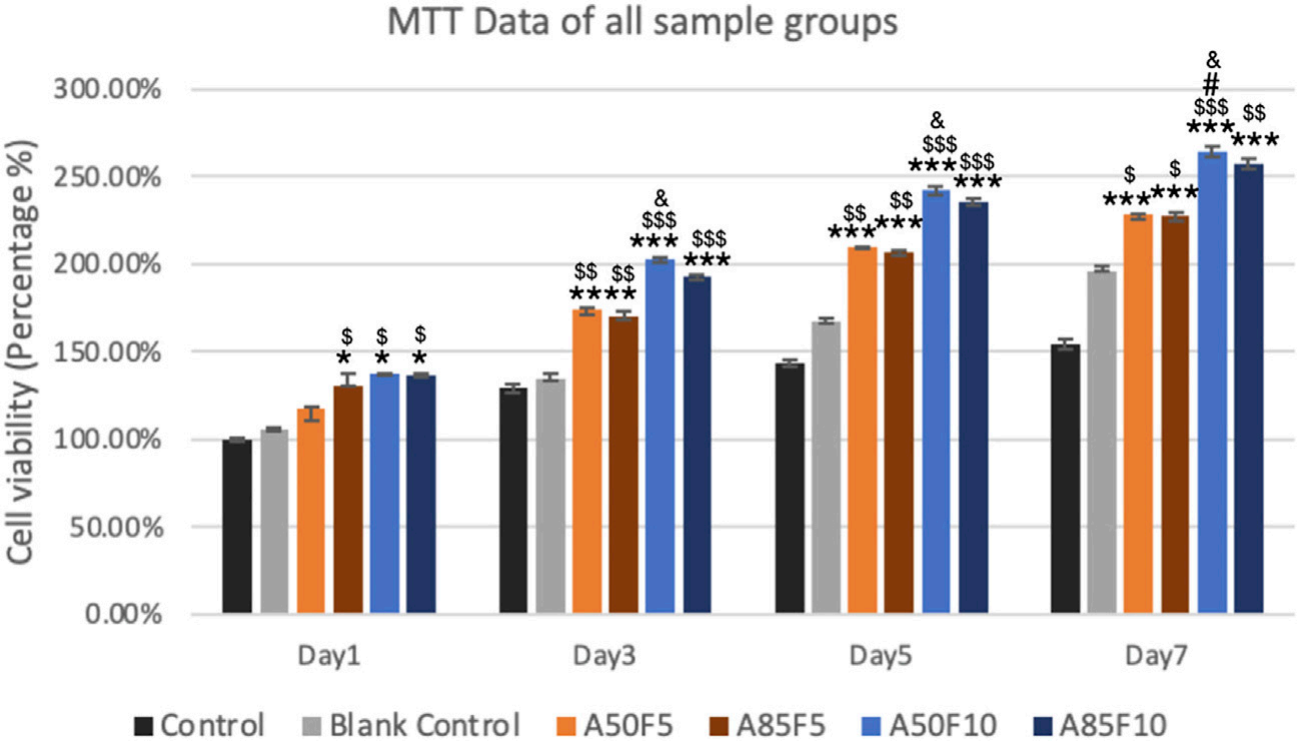
Increased cell proliferation and viability of surface treated samples especially fibronectin treated groups (blue lines) can be seen from day 1 to day 7 by using MTT analysis (Significant difference compared with control is indicated with *, *p* < 0.05, **, *p* < 0.005, and ***, *p* < 0.001, while $ symbol for comparing with blank control, # for A50F5 and, & symbol is applied for comparing with A85F5).

On day 7, A50F10 maintained the highest expression of cell viability compared with other groups. There was a statistically significant difference between all experimental groups and controls as well as between A50F10 and A50F5 and A85F5 groups (Figure 9).

### 3.8 Cell adhesion and proliferation by CLSM

**FIGURE 10:**
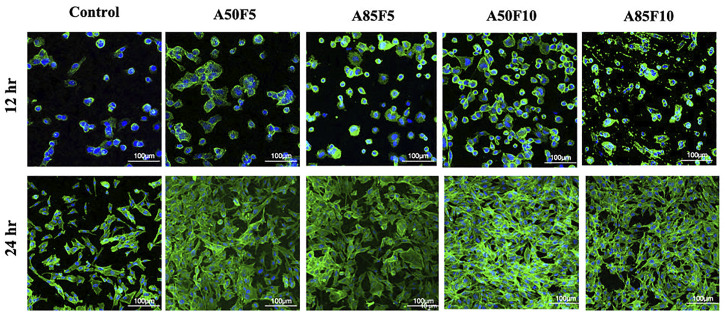
Confocal laser scanning microscopy (CLSM) images showing the cell proliferation on different surfaces of zirconia ceramic after culturing for 12 h and 24 h and demonstrating prominent cells adhesion and proliferation on 24 hrs.

At 12 h, MG-63 cells started to proliferate on the surface of zirconia and appeared to grow. Osteoblast-like cells were firmly attached to the Zr surface and continued to proliferate well at 24 h. Prominently appearing nuclei and F-actin filaments could be seen that become very dense for all groups as the surface become packed with cells (Figure 10). The A50F10 group had the largest number of cells followed by the A85F10 and A75F10 groups ***.***

### 3.9 Differentiation of osteoblast-like cells by ALP enzyme activity

**FIGURE 11:**
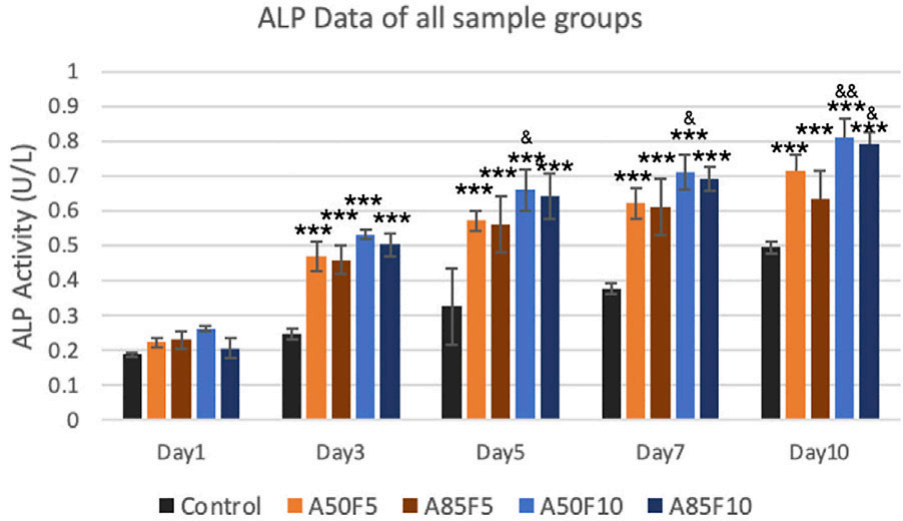
Surface modified samples of allylamine and fibronectin disks illustrated the increased ALP enzyme activity from day 1 to day 7 (Significant difference compared with control is indicated with *, *p* < 0.05, **, *p* < 0.005, and ***, *p* < 0.001 while # symbol for comparing with A50F5, & symbol is applied for comparing with A85F5).

ALP enzyme activity observed an upward trend, with the A50F10 disk at 0.8123 U/L showing the highest expression among all groups on day 7 and day 10. The A85F10 disk had the second highest result of 0.7911 U/L, followed by A50F5 at 0.7144 U/L on day 10. Statistically significant differences were present between the control and experimental groups from day 3 to day 10. Moreover, on day 5, day 7, and day 10, the A50F10 group had statistically significant enzyme activity compared to A85F5 (Figure 11).

### 3.10 Osteogenic markers by RT-qPCR

**FIGURE 12:**
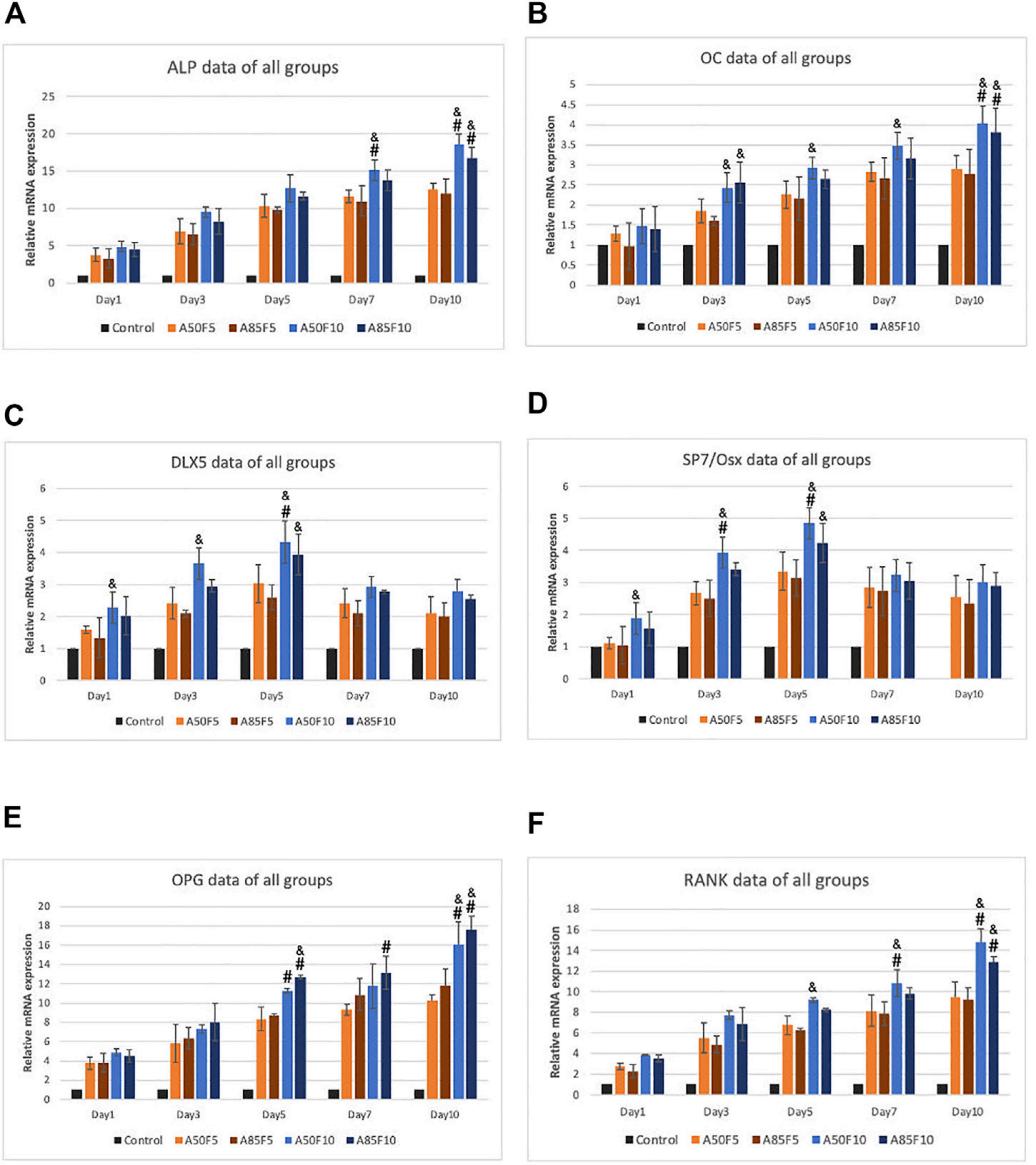
RT-qPCR data of all sample groups demonstrating increased upregulation of osteogenic related mRNA expression and A50F10 showed highest expression for **(A)** ALP, **(B)** OC, **(C)** DLX5, **(D)** SP7 and **(F)** RANK while A85F10 had highest expression for **(E)** OPG primers, (Significant difference compared with control is indicated with*, *p* < 0.05, **, *p* < 0.005, and ***, *p* < 0.001 while # symbol for comparing with A50F5, & symbol is applied for comparing with A85F5).

Relative gene expression of osteogenic mRNA markers including ALP, OC, DLX5, SP7/Osterix, OPG, and RANK was gradually elevated after cultivation (Figures 12A–F). The A50F10 group had the highest upregulation for all markers in day 7 and day 10, except for OPG in which A85F10 was most highly expressed. SP7 and DLX5 markers had early upregulation until day 5, though these began to downregulate on day 7 and day 10. For ALP and RANK, the A50F10 group had a statistically significant difference with F5 groups: A50F5 and A85F5 on day 7 and day 10 as well as the same result for DLX5 and SP7 on day 5. OC and OPG markers for A50F10 had a statistically significant difference with A50F5 and A85F5 groups only on day 10.

### 3.11 Mineralization by alizarin RedS staining

**FIGURE 13:**
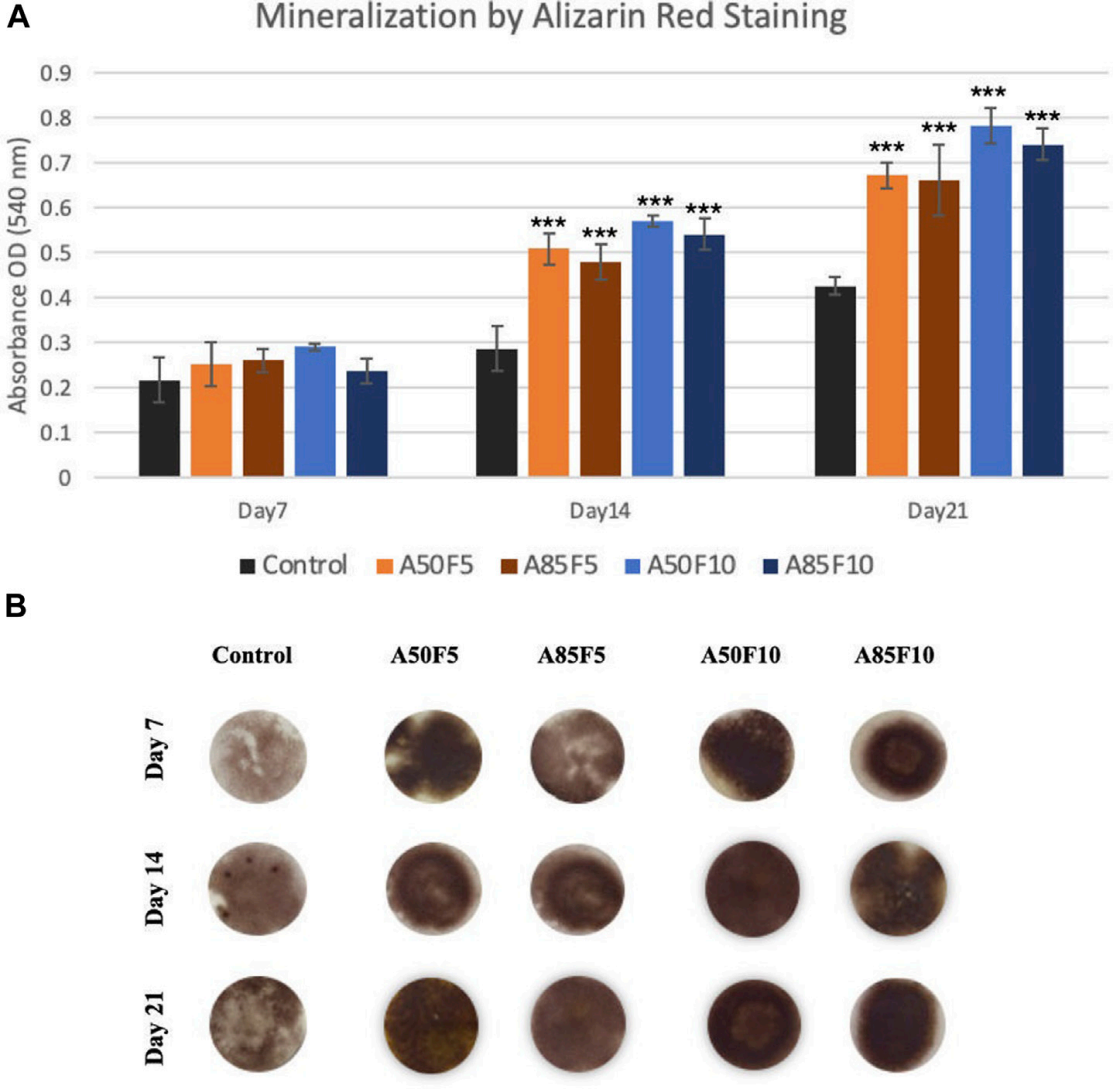
**(A)** Enhanced mineralization capability (late-stage markers) of osteoblasts on fibronectin grafted disks can be seen (Significant difference compared with control is indicated with *, *p* < 0.05, **, *p* < 0.005, and ***, *p* < 0.001, **(B)** Alizarin red S-stained images of sample groups from Day 7 to Day 21.

Osteogenic differentiation was investigated using alizarin red S staining. Figures 13A, B showed the qualitative analysis of calcium deposition in the extracellular matrix (ECM) after 7, 14, and 21 days of incubation in cell culture medium. A50F10 and A85F10 disks had the highest calcium composition at 14 and 21 days and were statistically significantly different (*p* < 0.001) from fibronectin grafted groups and the control disk in Figure 13B.

## 4 Discussion

In this study, physical and chemical analysis of surfaces were first conducted to confirm fibronectin attachment and were followed by testing biological characteristics. Irregularly folding fibronectin was observed on the surface and as the allylamine power and fibronectin concentration increased, the deposits become more prominent and abundant. Gradually increases in carbon and nitrogen as allylamine power increased were observed in XPS data (Table 2; Figure 4). This was because allylamine, an organic compound with the chemical formula C_3_H_5_NH_2,_ has abundant elemental carbon and nitrogen to donate (Gontard et al., 2014). As allylamine power increased from 50 W to 75 W and 85 W, the yellow surface yellow color darkened due to the surface becoming increasingly coated by allylamine and fibronectin. FTIR identified bonds and C-H, C≡C, and N-H functional groups for allylamine groups (Figure 5A) due to its organic chemical structure (C_3_H_5_NH_2_). GA has wide application in various fields as it possesses distinctive characteristics that make it one of the most efficient protein crosslinking reagents (Migneault et al., 2004). Alba Rodriguez-Nogales et al., found the NH_2_ group in silk fibroin nanoparticles, and after crosslinking with GA solution, N-O functional bonds were obtained (Rodriguez-Nogales et al., 2016). Traceable to the C_5_H_8_O_2_ chemical structure of GA in this study, N-O, C=O, C-H, and O-H bonds can be seen after GA crosslinking (Figure 5B), while peaks in the absorption band at 1,050, 1,360, 1,620, 2,700, and 3,200 cm^−1^ indicate the presence of C-O, N-O, N-H, C-H, and O-H functional bonds in all fibronectin samples because of the presence of COO-H and N-H functional groups in protein. Although some of the N-O functional bonds were covered by fibronectin proteins, some peaks for these absorption bands could still be detected. This FTIR spectra identified the chain reaction and cross-linking between GA solution and RGD peptides of fibronectin proteins. SEM, XPS, and FTIR data help confirm that fibronectin proteins have successfully grafted to zirconia samples, a finding consistent with *in vitro* and *in vivo* analysis of fibronectin conducted by Chang et al. (2016a). Fibronectin protein dots may spread with an inhomogeneous appearance in Figure 6A. Nevertheless, after careful calculation of the number of proteins present on the surface by fibronectin dots, there is no statistically significant difference in the amount of fibronectin proteins between inter-groups of F5 (A50F5, A75F5, and A85F5) as well as F10 inter-groups of (A50F10, A75F10, and A85F10) in Figure 6B. It indicated that the number of fibronectins attached to the surface was quite similar if the same concentration was used. However, there is a statistically significant difference data in between F5 and F10 groups. Possibly, longer fibronectin immersion time and increased plasma power setting can be tested in future studies to obtain a more homogeneous spreading manner of fibronectin on the surface.

Two more crucial tests for surface treatment samples are surface wettability and surface roughness. According to past studies by Schwarz F et al. and Parisi L et al., the surface of a biomaterial has an important influence on the differentiation of osteoblasts after changing their surface hydrophilicity (Schwarz et al., 2009; Chang et al., 2015; Parisi et al., 2020). After fibronectin treatment, a significant decline in contact angles of the disks (Figure 7A) was observed, implying that fibronectin coating can improve the affinity of cells and proteins to attach to the zirconia surface. In addition, there is a strong Pearson correlation coefficient of 0.763 between the contact angle and allylamine power and fibronectin concentration (Figure 7B). It should be noted that when treated with lower allylamine and fibronectin concentrations, lower contact angles and better hydrophilicity can be obtained. For surface roughness, rougher surfaces were observed to have higher fibronectin concentrations. *In vitro* experiments indicate that surface roughness can influence initial cell growth and metabolism, leading to improved clinical performance (Al Qahtani et al., 2017). A rough implant surface significantly enlarges the surface area, leading to faster osseointegration. In our study, we obtained a nanoscale surface roughness (Ra) value of 495 nm for A85F10 after surface modification while the original zirconia has a roughness of 114 nm. In addition, there is a moderate Pearson correlation coefficient (*r* = 0.56) between concentrations of allylamine, fibronectin, and surface roughness (Figure 8B). Thus, after treatment with fibronectin, increased surface roughness lead to faster osteoblast adhesion and bone apposition. Considering surface roughness and wettability in this study, increasing contact angles were observed with increased surface roughness. This data is also supported by Afida Jemat et al.’s comparison of 3 biomaterials which showed that increased contact angles can also be found with higher surface roughness (Jemat et al., 2018). Yu-Hua Pan et al., also showed in a comparison between control and GDP-treated zirconia disks that increased surface roughness is associated with higher contact angle (Pan et al., 2020). In the current study, the major objective was functionalization with fibronectin proteins to enhance osteoblastic-like cell adhesion and proliferation (Chang et al., 2016b; Liang et al., 2022) in combination with improved physical properties of surface roughness and wettability of zirconia ceramic.

Past studies have shown that the use of low-temperature GDP combined with allylamine treatment can modify the surface of biomaterials, and allylamine is a thin layer of positively charged amine-based nano-layer on the surface of the material (Nebe et al., 2019). Human osteoblasts have hyaluronic acid on their surfaces which has a net negative charge of −15 mV (Rebl, 2014), which is attracted to the positive electrical energy of the thin layer of acrylamide in nanometers, which is conducive to the attachment negatively charged cells. Indeed, our results confirmed that allylamine treated samples have better performance than the original Zr. However, after grafting with fibronectin 5 μg/mL (F5) and fibronectin 10 μg/mL (F10) in this study, remarkably better physical properties in terms of roughness and wettability can be seen over both control and allylamine grafted samples. After analyzing surface roughness and hydrophilicity data, four optimal groups (A50F5, A85F5, A50F10, and A85F10) were chosen for continued biological analysis.

2MTT assay detects the mitochondrial activity of cells by measuring tetrazolium salts and indirectly indicates cell viability and proliferation (van Meerloo et al., 2011). Figure 9 shows that MG-63 cells tended towards viability and proliferation on fibronectin-treated samples, with statistically significant cell proliferation compared with the control disk starting from day 1. On day 7, the group with the highest cell proliferation was A50F10, followed by groups A85F10 and A50F5. This result conforms with the increased cell proliferation data by an *in vitro* study of the effects of fibronectin protein on osteoblast cells, and *in vivo* analysis of fibronectin-modified titanium dental implants in dogs published by (Chatakun et al., 2014; Chang et al., 2015; Chang et al., 2016b). In this study, increased surface roughness and wettability of zirconia ceramic encouraged cell migration and adhesion properties of the surface coating. Thereby, leading to increased cell contact with the allylamine fibronectin combination and enhancing the cell viability and proliferation. A previous study by Chang et al., also found increased cell viability after 24 h of fibronectin grafting on titanium disks. A more recent study by Pan et al. showed that on day 5, plasma-treated Zr ceramic had significantly increased cell viability compared with other sample groups (Pan et al., 2020). MG-63 cells tended to proliferate more on the fibronectin-treated samples even after the first 12 h of culturing (Figure 10). Cells continued to grow well, adhering to and completely packing the surface by 24 h. The A50F10 sample demonstrated the highest cell proliferation at 12 h and 24 h, which also conforms with data obtained from MTT analysis. Calcium deposits and phosphatase activity, two classic indicators of osteoblast differentiation and bone mineralization, were evaluated. It was observed that the A50F10 and A85F10 groups showed increased ALP activity and calcium mineralization deposits compared to other surfaces (Figure 11 and 143. Other studies of surface coatings, such as those by Chen et al., showed similar results, demonstrating that after BMP-2 coating of Zr disks for 14 days, significant mineralization activity can be detected (Chen et al., 2022). A study by Pardun et al. showed that the ALP activity of cells on calcium phosphate-coated zirconia on day 7 and day 9 was significantly improved (Pardun et al., 2015).

Osteogenic-related mRNA expression of ALP, OC, DLX5, SP7/OS, OPG, and RANK was measured (Figure 12), with data matching the cell viability and differentiation. ALP is considered an early and commonly accessible osteogenic marker that indicates osteoblast differentiation (Hashemibeni et al., 2013; Rutkovskiy et al., 2016). OC is also produced by osteoblasts and is used as a marker for the bone formation. The fibronectin grafted sample A50F10 showed the highest and thus greatest gene expression for osteoblast cells. Interestingly, DLX5 and SP7 had early upregulation on day 1 and later downregulation on day 7 and day 10; this is because they are osteogenic markers of osteoblast differentiation at an early stage (Holleville et al., 2007; Yoshida et al., 2012). OPG and RANK work together in a signaling pathway that regulates osteoclast differentiation, activation, and bone remodeling (Boyce and Xing, 2007). Higher levels of OPG and RANK for fibronectin samples on Day 7 and Day 10 indicates the presence of significant bone remodeling—the cornerstone of new bone formation (Theoleyre et al., 2004). A previous study by Kang et al. showed upregulation of Runx-2 and OCN expression for fibronectin-stimulated stem cells on days 3 and 5 (Kang et al., 2017). Cho et al. also demonstrated that Zr with an aerosol deposition coating of hydroxyapatite improved ALP and OC expression (Cho et al., 2015).

Based on the above results, fibronectin-allylamine composite grafting significantly improved cell viability and proliferation without cytotoxicity compared with original zirconia disks. Allylamine is an organic compound with positive amine groups which attracts negatively charged biomolecules, thereby providing an ideal connection between the material and cells (Buddhadasa et al., 2018) and fibronectin is an ECM protein that binds to integrin and other ECM proteins such as collagen, fibrin, and heparan sulfate proteoglycans. Besides, its structure and composition regulate contextual cell signaling and have a major role in cell adhesion, proliferation, and differentiation (Patten and Wang, 2021) in addition to its function as integrin that allows cells such as fibroblasts or MG-63 cells to attach to a particular surface. Due to their positive benefits, allylamine and fibronectin compound is a prominent protein complex to coat in implant surface treatment research.

This study depended on the functions of bioactive proteins fibronectin and amine organic chemical reacting with surrounding bone cells to increase adhesion and proliferation. Though our findings on physical characteristics were not always in line with biological analysis, successful fibronectin attachment onto the ceramic surface also depend on combined physio-chemical properties of the surface thereby leading to improved cell viability and differentiation. Although surface improvement can be seen after allylamine-fibronectin treatment, we discovered that fibronectin played a more dominant role than allylamine compound in osteogenic cell responses. When fibronectin concentrations were higher, better results could be obtained; i.e., the F10 groups performed better than the F5 groups in biologic reactions with MG-63 cells. There is a significant difference in cell viability, differentiation, and osteogenic gene expression (ALP, OC, OPG, and RANK) between F5 and F10 concentration groups while A50F5 and A85F5 or A50F10 and A85F10 were not significantly different in MTT and qPCR result. From our study, biological analysis indicated that A50F10 and A85F10 can be regarded as the optimum conditions. Therefore, fibronectin proteins and allylamine combination additive procedures can be a reasonable consideration for future dental implant surface treatment applications.

Still, osteoconduction in fibronectin-grafted dental implants remains in question. Osteoconduction is the first and most important phase of healing, and relies on the recruitment and migration of osteogenic cells to an implant’s surface. Peri-implant blood clot residue and the initiation of platelet activation results in directed osteogenic cell migration (Weber, 2019). Miha Brezavšcˇek et al. demonstrated that photofunctionalization with UV treatment on zirconia-based material also enhanced the osteoconductivity (Brezavšček et al., 2016). In this study, fibronectin also assisted in the blood coagulation phase and encouraged the early recruitment of cells, which falls in agreement with the osteoconduction explanation*.* However, some limitations remain. As this study was performed *in vitro* study and no histological evidence of bone integration with the Zr was measured, we cannot prove the enhancement of osseointegration. As well, questions remain open regarding the efficacy of fibronectin functionalization in soft tissue healing, homogeneous spreading on surfaces, and strategies to improve the binding efficiency between zirconia ceramic surface, allylamine, and fibronectin interfaces. Commonly used reductive surface treatment methods, including sandblasting and/or acid etching techniques may pre-perform to increase the surface area and improve the binding efficacy between the surface coating and zirconia ceramic surface. In addition, the power of plasma allylamine treatment and fibronectin immersion time setting for protein grafting may be adjusted to longer periods for better affinity between coating and zirconia. Further study research and discussion into the functionalization of zirconia ceramic with fibronectin proteins is required.

## 5 Conclusion

Surface modification using glow discharge plasma combined with allylamine and fibronectin proteins composite significantly improved the physical and biological properties of zirconia biomaterials. Surface hydrophilicity and roughness were greatly improved after surface modification of zirconia ceramic. Most importantly, allylamine-fibronectin grafting enhanced its bio-activity on osteoblasts. It improved the cell-material interaction, overcoming the inherent bio-inertness of zirconia, as evidenced by the enhanced viability, proliferation, differentiation, and upregulation of osteogenic-related mRNA of osteoblast-like cells which play a major role in bone formation and remodeling. A50F10 showed the optimal result in this study, and we recommend additional research into the utilization of fibronectin coating for future dental implant applications.

## Data Availability

The original contributions presented in the study are included in the article/supplementary material, further inquiries can be directed to the corresponding authors.
